# One-shot intervention reduces online engagement with distorted content

**DOI:** 10.1093/pnasnexus/pgaf068

**Published:** 2025-03-04

**Authors:** Eeshan Hasan, Gunnar Epping, Lorenzo Lorenzo-Luaces, Johan Bollen, Jennifer Sue Trueblood

**Affiliations:** Department of Psychological and Brain Sciences, Indiana University, Bloomington, IN 47405, USA; Cognitive Science Program, Indiana University, Bloomington, IN 47405, USA; Department of Psychological and Brain Sciences, Indiana University, Bloomington, IN 47405, USA; Cognitive Science Program, Indiana University, Bloomington, IN 47405, USA; Department of Psychological and Brain Sciences, Indiana University, Bloomington, IN 47405, USA; Cognitive Science Program, Indiana University, Bloomington, IN 47405, USA; Luddy School of Informatics, Computing, and Engineering, Indiana University, Bloomington, IN 47405, USA; Department of Psychological and Brain Sciences, Indiana University, Bloomington, IN 47405, USA; Cognitive Science Program, Indiana University, Bloomington, IN 47405, USA

**Keywords:** social media, mental health, depression, cognitive behavioral therapy, large language models

## Abstract

Depression is one of the leading causes of disability worldwide. Individuals with depression often experience unrealistic and overly negative thoughts, i.e. cognitive distortions, that cause maladaptive behaviors and feelings. Now that a majority of the US population uses social media platforms, concerns have been raised that they may serve as a vector for the spread of distorted ideas and thinking amid a global mental health epidemic. Here, we study how individuals (n=838) interact with distorted content on social media platforms using a simulated environment similar to Twitter (now X). We find that individuals with higher depression symptoms tend to prefer distorted content more than those with fewer symptoms. However, a simple one-shot intervention can teach individuals to recognize and drastically reduce interactions with distorted content across the entire depression scale. This suggests that distorted thinking on social media may disproportionally affect individuals with depression, but simple awareness training can mitigate this effect. Our findings have important implicasstions for understanding the role of social media in propagating distorted thinking and potential paths to reduce the societal cost of mental health disorders.

**Significance Statement**

Depression is marked by cognitive distortions—unrealistic, negative thoughts. Concerns have grown that social media may play a role in the development of depression, potentially through the diffusion of content that expresses cognitive distortions on social media. We use a simulated social media environment to test how individuals with varying degrees of depression interact with distorted content. We find that individuals with higher depression symptoms are indeed more likely to engage with and distribute distorted content. However, a simple one-shot intervention can teach individuals to recognize and reduce their interactions with such content by up to 50% in some cases. These findings highlight how social media may impact mental health through the diffusion of distorted content, suggesting that awareness training can mitigate these effects.

## Introduction

There are worrying indications that the prevalence of internalizing disorders such as depression has increased over the past decades (1), reaching levels that some have referred to as a mental health epidemic. Depression is, however, treatable. Cognitive-behavioral therapy (CBT) is the gold-standard psychological treatment for depression and anxiety (2–4). CBT is based on the tenet that cognitive distortions i.e. exaggerated, overly negative, and unrealistic ways of thinking that arise automatically and with minimal reflection, give rise to maladaptive behaviors and psychopathology. (See Table 1 or Supplementary material for more examples.) CBT therapists use psychoeducation, modeling, and skills-building to guide individuals to recognize and modify their distorted thoughts (4). For example, a person expressing the thought “I’ll never find love” might be exaggerating difficulties in finding a romantic partner, leading to hopelessness and social withdrawal. By reshaping this thought into “I have not found love yet but may meet someone if I put myself out there,” an individual may find it easier to acknowledge their challenges while maintaining hope.

**Table 1.** Examples of distorted and nondistorted tweets used in our experiment of varying VADER sentiment.

Type	Tweet	VADER composite score	Rank out of 30
Distorted	I’ll never find love. I’m just not good enough for anyone.	−0.70	5
Distorted	I’ll never be able to handle the morning rush hour. I’m just not a good enough driver.	−0.34	15
Distorted	Finances are tight and I’ll never be able to get ahead.	0.00	25
Nondistorted	The new phone I just bought is disappointing. Its slow, the battery drains quickly and the camera is subpar.	−0.49	5
Nondistorted	This weather has been stormy. It’s been raining nonstop for days and I do not like it.	−0.28	15
Nondistorted	Bad luck is a part of life, but it’s important to persevere.	0.24	25

The sentiment rank was calculated separately for distorted tweets and nondistorted tweets using the VADER scores. Low rank and low VADER sentiment score indicate negative sentiment.

The CBT model is well-supported by clinical–translational and experimental research that finds that cognitive vulnerabilities are predictive of depression as well as suggesting that cognitive reappraisal, the ability to look at situations from different perspectives, is the most efficacious emotion regulation strategy (5, 6). However, CBT inevitably takes place in an individual’s psychological, social, and cultural context which is in our current context marked by the ubiquity of social media: over 70% of US adults are on a social media platform and current generations report being on social media “constantly.” Commentators have linked a recent increase in depression, especially among youth, with the rise of social media (e.g. (7)), with speculations that social media with its bite-sized content, rapid spread of information, and fast spread of negativity may promote a type of depressive thinking.

There are strong indications of a bidirectional association between depression (and other internalizing disorders) and social media use (8–15). First, research indicates that having depression is associated with distinct and recognizable behaviors on social media. Compared to healthy controls, individuals with depression exhibit different temporal usage patterns (8), use distinct language (9), and express higher levels of cognitive distortions (10, 16). These findings are further supported by the performance of machine learning methods that produce diagnostic evaluations by recognizing linguistic markers in the online language of depressed individuals (see Ref. (17) for a recent review). Second, social media use itself is associated with depression as highlighted in various studies (11–15). The mechanism for social media use as a risk factor for depression is unclear, but since much social media use involves consuming content, exposure to and interaction with some types of social media content may increase the affective, behavioral, and cognitive risk factors for depression. Some “induced rumination” research indeed supports that exposure to sources similar to social media content can worsen mood and increase depression symptoms (18–21).

Since social media could be involved in the diffusion of content expressing cognitive distortions, this could be a matter of great importance to billions of individuals. Research indicates the potential for social mood contagion (22–24), possibly as a result of affect assortativity in social networks (25–28) modulated by language features such as moral–emotional words (29, 30). However, the same potential for social contagion on social media could in fact be leveraged to mitigate its possible deleterious effects.

In fact, beyond vectors for social contagion, social media platforms may also offer a unique opportunity for low-cost interventions that, relative to therapeutic approaches like individual CBT, can be more scalable and yield large cumulative or compounding effects across a social network. These approaches have been shown to be effective in the related area of online misinformation. Individuals can be taught critical thinking skills to distinguish biased and fallacious claims (31). Warning and educating individuals against misinformation can serve as an effective “inoculation” strategy to mitigate its online diffusion (32). In fact, individuals share less misinformation when they are asked to reflect on the accuracy of statements (33). Similar approaches could be leveraged to “boost” individuals to have more healthy interactions via social media (34) since existing research supports the efficacy of CBT-based microinterventions (35), including interventions that leverage social media to teach CBT principles (36).

**Fig. 1.**
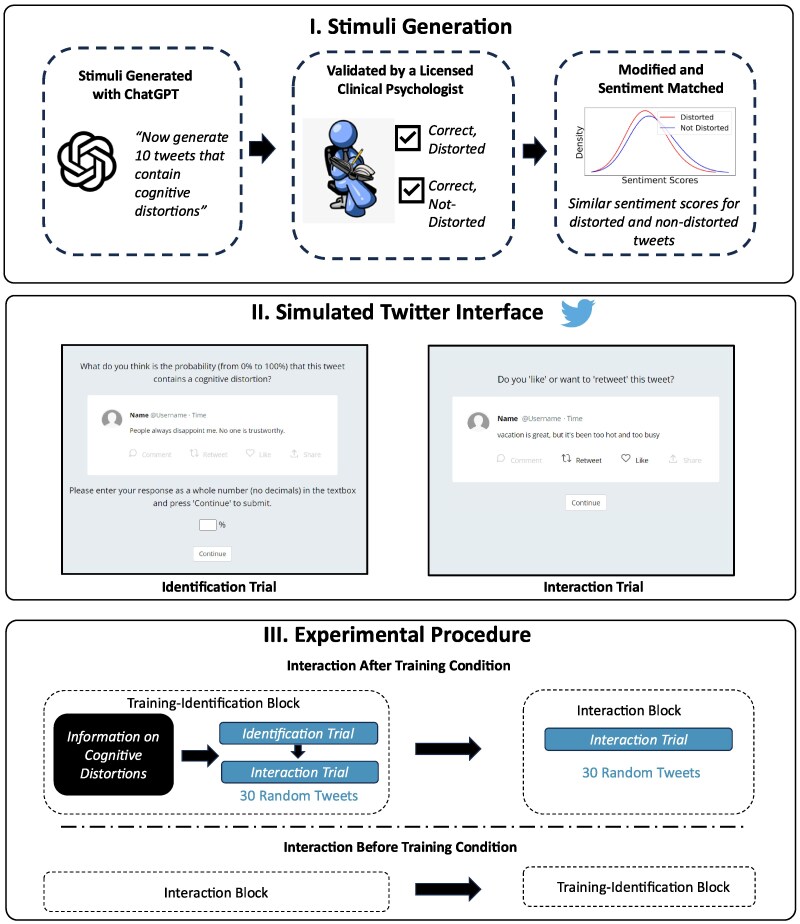
Study design. The top panel depicts the four-step stimuli generation procedure: (i) large language model stimulus generation, (ii) clinical validation, (iii) sentiment analysis, and (iv) final validation. The middle panel depicts the identification and interaction trials in our simulated social media environment. The bottom panel depicts the randomization and experimental procedure. Participants who were assigned to the “Interaction After Training Condition” did the Training-Identification block before the Interaction Block and the individuals in the “Interaction Before the Training Condition” did it in the opposite order. Image in the top panel used from the clipart library: https://clipart-library.com/clipart/1682104.htm.

Given the potential of mitigating the possible effects of exposure to cognitive distortions on social media, we designed an experiment to study how individuals with varying levels of depression engage with distorted social media content and to test the effects of a simple one-shot intervention that educates social media users about cognitive distortions. We presented individuals with a set of curated tweets in a simulated Twitter (now X) environment and observed the rates at which they (i) recognize and (ii) like and share distorted content vs. a control set of tweets that carry a similar sentiment. As shown in Fig. 1, we produce the tweets with a large language model (GPT-3) accessed through ChatGPT with minimal context so that the tweets generated were independent of experimenter biases in content and linguistic style (see Table 1 or Supplementary material for examples). These tweets were further sentiment-matched, i.e. for each tweet with a cognitive distortion, we ensured there existed a counterpart without a cognitive distortion, but similar sentiment. All tweets were validated by a licensed clinical psychologist with extensive expertise in CBT.

To ensure ecological validity in our study, we created an interface similar to what users of the social media platform Twitter (now X) would see when they read a “tweet,” as shown in Fig. 1. Participants were exposed to individual tweets and provided interactive buttons that lit up upon being “liked” or “retweeted,” much like the Twitter or other social media interfaces (37, 38).

We trained individuals to identify distorted language with a short psychoeducational intervention. The training consisted of two parts. Initially, participants were presented with a brief overview of cognitive distortions along with illustrative examples. Next, they were asked to evaluate the probability of different tweets being distorted before deciding to like or retweet them. This process was designed to encourage participants to consider the possibility of distortion in a statement before interacting with it. Importantly, the training did not explicitly address the harmful aspects of cognitive distortions, nor did it advise participants against engaging with such content.

We aimed to assess how training influences individuals’ interactions with social media content by studying their interactions before and after they received the training, as depicted in the bottom panel of Fig. 1. We therefore established two distinct conditions in the experiment by manipulating the order of the training-identification block and the interaction block. In one condition, participants interacted with tweets, by liking or retweeting. In the other condition, the same interactions were performed after the participants had completed the training. The primary objective of the experiment was to test if individuals could be trained to detect distorted social media content and how this training impacted their behavior in terms of the kind of content they engaged (“liking” or “retweeting”). The secondary objective was to understand recognition and interaction behavior at differing levels of depression. Specifically, to study (i) how individuals with differing levels of depression interacted with distorted social media content, (ii) if individuals across the depression scale can be trained to identify distortions, and (iii) if the training changed the behavior of individuals with differing levels of depression vs. a control group of people who were not similarly trained and at different levels of depression indicators (including none).

## Results

We used the Patient Health Questionnaire-9 (PHQ-9) a brief self-report scale to measure the depression severity (39). The questionnaire asks individuals questions about the frequency of symptoms of major depression (e.g. worthlessness, weight gain, insomnia) during the preceding 2-week period. This questionnaire has been shown to have a high specificity and sensitivity for measuring depression severity (39–41).

We used self-report measures to calculate an aggregated Twitter Use Score (TUS). Specifically, individuals reported the number of hours they spend on Twitter, the ratio of their active and passive Twitter use, and their willingness to pay for Twitter. These questions were used to estimate the TUS where a greater score indicates more involvement with Twitter. For the distribution of these individual measures, see Fig. S2. The full details of the score calculation are presented in the Methods section.

### Liking and retweeting

In our simulated Twitter environment, participants engaged with tweets by either liking or retweeting them. We studied the relationship between depression and the distortedness of the content by focusing on the condition where individuals interacted with the tweets before training. We observed that the depression levels were positively associated with the like (ρ(416)=0.23,P≤0.001) and retweet rates (ρ(416)=0.22,P≤0.001). Further, we observed that more depressed individuals exhibited a higher tendency to like (ρ(416)=0.34,P≤0.001) and retweet (ρ(416)=0.24,P≤0.001) distorted content. Together, our findings indicate that depressed users not only tended to like and retweet tweets with distorted content but also were more likely to interact with tweets in general.

We conducted a generalized mixed-effects regression analysis (see Table 2) to further understand how distortedness, depression severity, social media usage, and their interactions relate to engagement with social media content. We treated item and participant as random effects to account for item-level effects and individual idiosyncrasies.

**Table 2.** Results of generalized mixed effects regression predicting accuracy, liking, and retweeting, from Tweet distortion, user depression, training block, and their interactions (n=838).

	*Dependent variable*		
	Accuracy	Like	Retweet
Age	1.068	1.239*	1.288*
	[0.944,1.209]	[1.051,1.460]	[1.032,1.607]
Woman	1.274**	1.096	0.832
	[1.089-1.490]	[0.889,1.352]	[0.630,1.099]
Nonbinary	1.774*	0.991	0.626
	[1.095,2.872]	[0.528,1.859]	[0.269,1.458]
Interaction after training	0.941	1.035	1.045
	[0.787,1.125]	[0.840,1.273]	[0.794,1.375]
Is distorted	0.342***	0.212***	0.326***
	[0.236,0.495]	[0.133,0.337]	[0.214,0.496]
Depression severity	0.205***	1.613	4.187***
	[0.124,0.339]	[0.875,2.973]	[1.878,9.334]
Twitter Use Score	0.152***	1.742*	6.190***
	[0.097,0.237]	[1.016,2.986]	[3.039,12.610]
Interaction after training:is distorted	1.093	0.553***	0.424***
	[0.944,1.266]	[0.475,0.645]	[0.343,0.524]
Interaction after training:depression severity	1.528	2.024	1.123
	[0.737,3.168]	[0.836,4.899]	[0.353,3.569]
Is distorted:depression severity	6.482***	7.243***	5.135***
	[4.303,9.763]	[4.884,10.740]	[3.177,8.300]
Interaction after training:Twitter Use Score	0.657	0.600	0.346*
	[0.343,1.260]	[0.275,1.309]	[0.124,0.968]
Is distorted:Twitter Use Score	3.831***	3.640***	3.327***
	[2.671,5.494]	[2.528,5.241]	[2.136,5.181]
Interaction after training:is distorted:depression severity	0.789	0.986	1.158
	[0.431,1.443]	[0.537,1.810]	[0.526,2.552]
Interaction after training:is distorted:Twitter Use Score	1.326	1.210	2.046
	[0.781,2.251]	[0.698,2.100]	[0.993,4.217]
Constant	7.698***	0.347***	0.064***
	[5.222,11.348]	[0.210,0.574]	[0.035,0.115]
Observations	25,140	25,140	25,140
Log likelihood	−10,674.320	−11,054.830	−7,345.849
Akaike Inf. Crit.	21,382.650	22,143.660	14,725.700
Bayesian Inf. Crit.	21,520.900	22,281.910	14,863.940

* P<0.05; ** P<0.01; *** P<0.001 Item and participant were treated as random effects. Income, PHQ-9, and TUS were range normalized (scaled to a 0 to 1 range). Logistic regression coefficients were exponentiated to allow odds ratio interpretations, representing the change in odds for each unit increase in the predictor. The 95% confidence intervals are shown in the rows below the coefficients.

More depressed individuals engaged with distorted content more than nondistorted content compared to less depressed individuals, as indicated in the left panels of Fig. 4. This was further corroborated by the significant interaction with depression severity and distortedness in predicting liking ($e^{\beta }=7.243$, 95% CI = [4.884, 10.740]) and retweeting ($e^{\beta }=5.135$, 95% CI = [3.177, 8.300]). For nondistorted content, individuals across the depression severity scale liked content at a similar rate, as indicated by the nonsignificant coefficient of depression severity for predicting liking ($e^{\beta }=1.613$, 95% CI = [0.875, 2.973]). However, individuals with greater depression severity retweeted nondistorted content at a higher rate ($e^{\beta }=4.187$, 95% CI = [1.878, 9.334]). Thus, we find that individuals with greater depression severity retweet all content (both distorted and nondistorted) at higher rates (see Fig. S4). Additionally, greater depression severity is correlated with increased liking and retweeting of distorted content as compared to healthier individuals.

Since individuals with high Twitter involvement are potentially exposed to distorted language (29, 30) even if they are not depressed, we tested how participants with higher Twitter involvement (as indicated by their “TUS”) interacted with distorted language in our simulated environment. We observe that a higher TUS was associated with increased liking and retweeting (like: ρ(416)=0.18,P<0.001, retweet: ρ(416)=0.18,P<0.001) (see Fig. S4). TUS significantly predicted the liking rate ($e^{\beta }=1.742$, 95% CI = [1.016, 2.986]), and the retweeting rate ($e^{\beta }=6.190$, 95% CI = [3.039, 12.610]). As shown in the right panels of Fig. 4, higher TUS predicted an increased tendency to interact with distorted content. This was corroborated by the interaction between TUS and distorted content in predicting the like and retweet rates (liking: $e^{\beta }=3.640$, 95% CI = [2.528, 5.241], retweeting: $e^{\beta }=3.327$, 95% CI = [2.136, 5.181]). This indicates a higher tendency for individuals with high TUS to interact with distorted content.

### Training individuals to detect cognitive distortions

Individuals were trained to detect cognitive distortions using a short document of less than 250 words which was adapted from an online CBT intervention and developed to be easily understood by participants (provided in the Supplementary material). As shown in Fig. 1, individuals were asked to identify if a tweet contained a cognitive distortion for 30 randomly chosen tweets. Since it was sometimes not possible to judge whether a statement was distorted without additional context, participants judged the probability that a statement contained a cognitive distortion. We report the performance on two metrics—accuracy and slope. The accuracy was calculated by binarizing their probability judgments at 50 and calculating the fraction of correct responses. The slope was calculated by subtracting the mean probability judgment on the distorted items from the mean probability judgment on the nondistorted items (42).

Despite the short training, most participants learned to detect cognitive distortions with high accuracy (mean: 0.791, SD: 0.158, median: 0.833, IQR: 0.708−0.900). The slope metric shows that they provided probability judgments that distinguished between distorted and nondistorted content (mean: 0.507, SD: 0.278, median: 0.590, IQR: 0.362−0.718). We note that accuracy and slope were highly correlated at ρ(836)=0.86,P<0.001, indicating that these measures were measuring similar abilities (see Fig. S1).

**Fig. 2.**
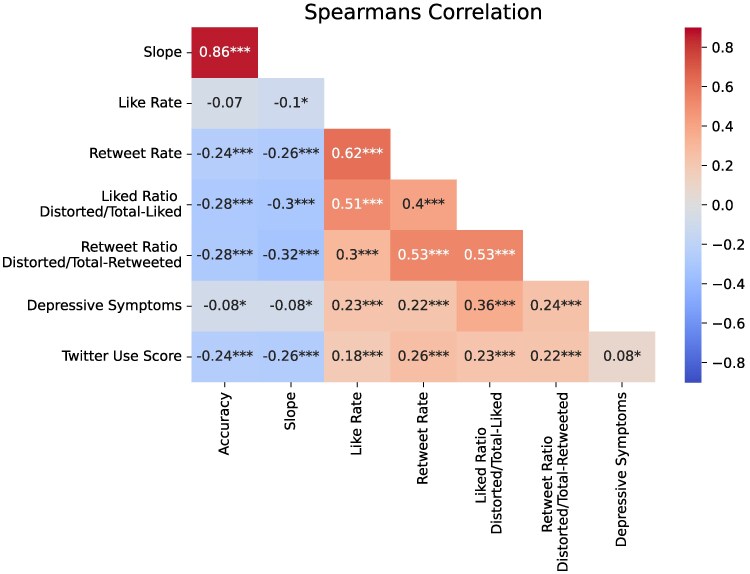
Correlations between the different measures of interest (n=838). Variables involving liking and retweeting only use the participants assigned to the interaction before the training condition (n=418). Three key observations are (i) Depressive Symptoms are weakly correlated with accuracy, (ii) Twitter score is moderately negatively correlated with accuracy, and (iii) Depressive Symptoms and TUS are moderately positively correlated with a higher ratio of liking and retweeting of distorted content to total content. The number of stars indicate the significance *P<0.05, **P<0.01, ***P<0.001.

We observed weak negative Spearman rank correlations between depression severity and accuracy and slope (accuracy: ρ(836)=−0.08;P=0.0167; slope: ρ(416)=−0.08;P=0.0181), as shown in Fig. 2 (see Fig. S3). We interpret this as a significant but small effect (43, 44), indicating that individuals with higher levels of depression severity learned the distortion identification task at similar but slightly lower levels as compared to healthier participants.

As shown in Table 2, individuals with greater depression severity have lower accuracy on the nondistorted items ($e^{\beta }=0.205$, 95% CI = [0.124–0.339]). However, those with greater depression severity have higher accuracy on the distorted items ($e^{\beta }=6.482$, 95% CI = [4.303–9.763]). This indicates that individuals with greater depression severity are more likely to report that items are distorted rather than nondistorted compared to healthier individuals.

Individuals with higher levels of Twitter engagement were worse at distinguishing distorted and nondistorted language, as indicated by the negative correlation between TUS and accuracy: ρ(836)=−0.24;P≤0.001 and slope: ρ(836)=−0.26;P≤0.001 (see Fig. S3). We also observed a negative main effect of TUS on accuracy ($e^{\beta }=0.152$, 95% CI = [0.097–0.237]) but a positive interaction with distorted items ($e^{\beta }=3.831$, 95% CI = [2.671–5.494]), indicating that individuals with a higher TUS have a higher tendency to report that items are distorted.

### Impact of intervention

**Fig. 3.**
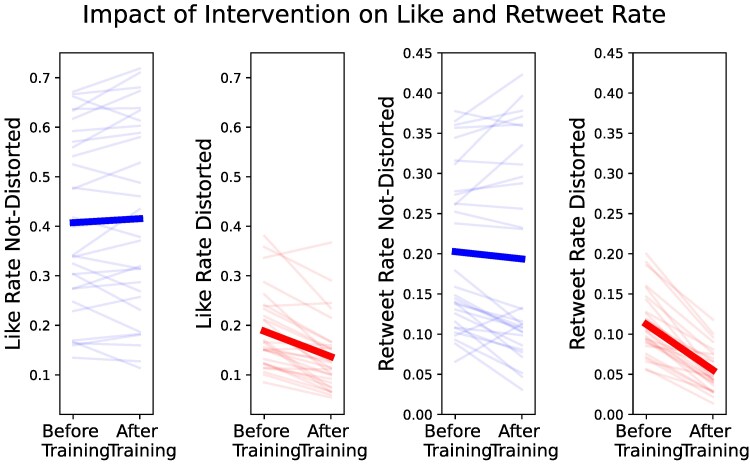
The impact of cognitive distortion psychoeducation on liking and retweeting distorted and nondistorted content. The thick dark lines depict the mean tendency. The light lines depict separate statements in the experiment. We observe a consistent reduction in the liking and retweeting of tweets with distorted content after the intervention compared to before the intervention.

Our training intervention reduced engagement with distorted content while not impacting engagement with nondistorted content (see Fig. 3). After training, we observed a sharp decline in the mean like rate (from 18.9% to 13.7%) and in the mean retweet rate (from 11.2% to 5.5%) for distorted items. For the nondistorted items, we observed no change in the mean like rate (from 41.8% to 41.2%) and the mean retweet rate (from 20.4% to 19.3%) after training.

These observations were corroborated by a generalized mixed-effects regression analysis predicting like and retweet rates based on the timing of training and the classification of items as distorted or not (see Table 2). For nondistorted content, the liking and retweeting rates were independent of whether the interaction was before or after the training as indicated by the null effect of the block order (like $e^{\beta }=1.035,95\%\;\text{CI}=[0.840,1.273]$, retweet $e^{\beta }=1.045$, 95% CI = [0.794–1.375]). However, there was a significant decrease in the liking and retweeting rates of distorted content when the interactions followed training (like: $e^{\beta }=0.553$, 95% CI = [0.475–0.645], retweet: $e^{\beta }=0.424$, 95% CI = [0.343–0.524]).

**Fig. 4.**
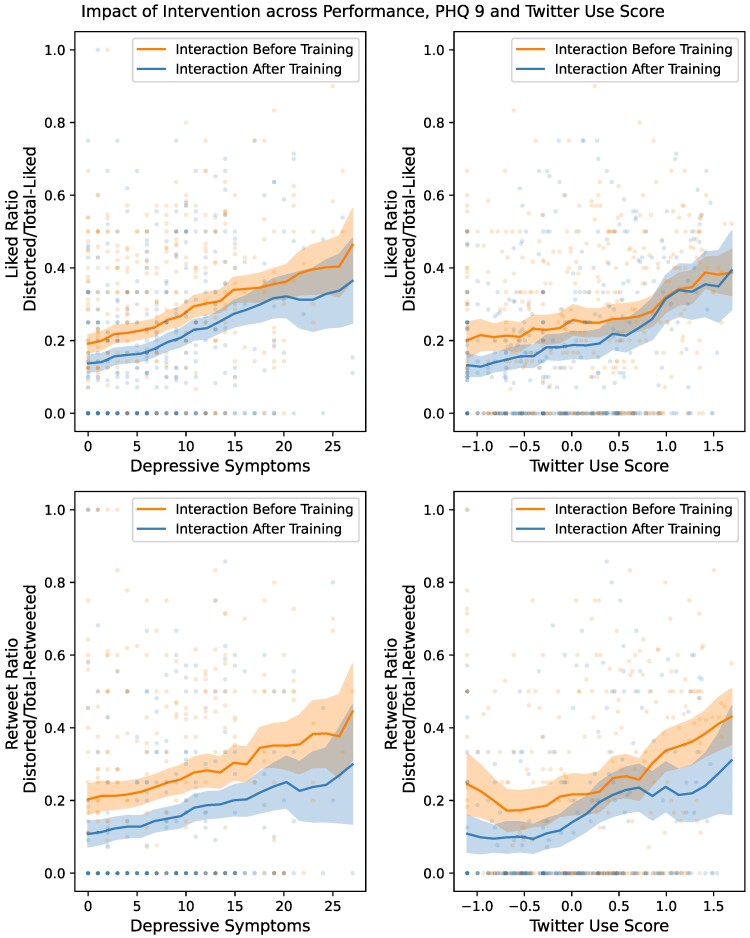
The impact of cognitive distortion psychoeducation across depression severity, and TUS (n=838).

We also find that the training effectively reduced both liking and retweeting of distorted content across different levels of depression (as shown in the left panels of Fig. 4). This indicates that the decrease in liking and retweeting due to training did not depend on the depression severity when compared to liking and retweeting without training. We further validate this statistically by observing a null relationship between how liking and retweeting depended on the three-way interaction between timing of interaction, distortedness, and depression severity for liking and retweeting; like: $e^{\beta }=0.986$, 95% CI = [0.537–1.810] retweet: $e^{\beta }=1.158$, 95% CI = [0.526–2.552]).

### Robustness checks

In the previous sections, many of our inferential statistics were based on the generalized mixed effects regression. In the Supplementary material, we test many other functional forms such as independent and interactive treatment of depressive severity and TUS and show that the results are robust to the different assumptions made by different models. (See Tables S1–S3 for regression coefficients and Table S4 for the nested model comparison using the likelihood ratio test.)

## Discussion

We conducted a large online study to better understand how individuals interact with distorted content online. We observed the effects of depression on interactions with content and whether training individuals to identify distorted content changed their (simulated) interactions. First, individuals with greater depression severity interacted with distorted content more than healthier individuals. Second, a psychoeducational microintervention that trained individuals to detect cognitive distortions was very effective in teaching individuals to distinguish distorted language from nondistorted language. Third, there was only a small difference in learning across depression severity levels. Fourth, the training sharply reduced the like rate for distorted content by around 30% and the retweet rate for distorted content by around 50%. Finally, this intervention reduced the sharing propensity across the depression severity scale. Thus, we find that a short “one-shot” psychoeducational intervention can substantially change the way individuals interact with and, perhaps more importantly, share distorted content on social media.

We stress that our psychoeducational intervention did not mention the link between distorted thinking and mental health disorders nor did we caution participants about sharing distorted content on social media. Similar to the accuracy prompt (33), the training goal was to improve an individual’s ability to identify distorted content. Our results show that even a short single-session educational training could improve the ability of individuals to recognize distorted content and change their interactions with such content.

We observed that regardless of the depression severity, individuals learned to distinguish distorted content from nondistorted content. For individuals with greater depression severity, we observe higher accuracy on the distorted items compared to the nondistorted items. This suggests that the reason depressed individuals produce (10) and interact with distorted content is not because they are unable to identify the distortion. Instead, we see a greater tendency towards reporting that an item is distorted. Similar to confirmation bias and motivated reasoning, where individuals are more likely to believe and endorse content that aligns with their beliefs and goals (45–47), depressed individuals may be more likely to endorse or share social media content that aligns with their distorted beliefs.

Our results might support a better understanding of how network dynamics interact with mental health on social media platforms. Previous studies have shown that online social networks are assortative both in terms of politics and affective-emotional states (25–27). That is, individuals with similar affective-emotional states tend to be connected, a well-known homophillic effect in network theory (48). In the case of social media, it is however unclear whether the observed assortativity is due to individual or algorithmic factors (28). For this reason, our simulated environment prompted each individual with the same set of curated tweets to equalize possible algorithmic exposure effects. Our results indicate that the previously observed assortative dynamics may be partly due to depressed individuals endorsing distorted content produced by other depressed individuals, thereby fostering social connections and causing a “birds of a feather”-effect with yet unknown psychosocial effects. The impact of our psychoeducational intervention demonstrates that being aware of cognitive distortions can however reduce endorsement and sharing rates of distorted content. The outcomes of such interventions may vary across different social media platforms. Future work could investigate whether our training might help individuals on content-based platforms like TikTok to identify and unfollow creators with distorted content. In community-based platforms such as Reddit, our training might help individuals leave communities with distorted content. Clinical therapeutic interventions might be tailored to social media behavior to help individuals with depression better navigate their social media landscapes and potentially avoid network effects that could present risk factors for cognitive distortions and internalizing disorders.

We observe that despite our training, the level at which individuals with greater depressive severity express distortions does not come down to the levels of healthier participants. There might be important reasons why individuals with depressive symptoms might express distorted thoughts on social media despite awareness. For instance, these might connect individuals who help and support each other in their mental illness (49). However, even relatively benign expressions of distorted humor can have negative social consequences (50).

We also observed that individuals who reported increased Twitter involvement (i) liked and retweeted distorted content at higher rates and (ii) were worse at identifying distorted content. These individuals also had a higher tendency to report that items were distorted. This might be partly explained by a “desensitization effect” from exposure to similar content on social media (29, 30).

### Constraints on generality

There are several important constraints to the generality of our results. First, our results might be specific to Twitter, the social media platform we tried to emulate, or other microblogging platforms, since shared content might be different across social media platforms (51). However, we study engagement behavior, such as likes and reposts, which are common across many different social media platforms. Further, we used simulated interactions without consequences, thus caution should be exercised in drawing generalizations about real-world social media interactions. This approach however gave us experimental control which addressed confounding issues such as possible differential exposure to distorted content in depressed individuals’ social media timelines (52, 53). Experimenting with real-world interactions by manipulating the distortedness of social media content (28) or using bots (e.g. (54)) could have adverse effects and must be done with stringent ethical oversight.

We used the PHQ-9 questionnaire to assess depression and its severity (39). We chose the PHQ-9 since it could be self-administered and was relatively short, allowing us to sample a large number of individuals and conduct a high-powered experiment. However, the PHQ-9 can report a false positive for related mood disorders, like bipolar disorder, leading to a low specificity in specific settings like a psychiatric hospital (55). We caution the reader that our scale might better be characterized as a scale of depressive symptoms rather than clinical depression (55). However, several meta-analyses that evaluated the PHQ-9 against the gold standard of clinical depression diagnosis have shown that the scale has a high sensitivity and specificity against being able to detect major depressive disorder (39–41).

### Future directions

Our study focused on the effects of a scalable, “one-shot” training to identify distortions in online content. We tested the effectiveness of this approach by observing interactions immediately after the psychoeducational intervention. In the future, one might design studies to study the long-term effects of such an intervention. In some cases, single-shot training might have long-term impacts. For instance, dialogs with a generative AI model increased the long-term impacts of a single interaction (56) to counter conspiratorial beliefs. However, a study that looked at a single-shot intervention on the principles of cognitive-behavioral therapy did not find lasting impacts on depression and anxiety symptoms several weeks after the intervention (57). Similarly, the effects of training individuals to identify online misinformation decayed over time (58, 59). The lack of longer-term effects might be countered by repeated boost sessions (60). Future studies should address this knowledge gap by studying the impact of short-term single-shot interventions over extended periods and ways to extend these effects with boosting interventions.

Our training intervention was effective in the context of our experiment, but it was oriented towards “recognition” of cognitive distortions in short social media posts, which does not imply a direct link with decreased depression over time. Future research might test the mediating impact of increased detection and reduced interaction on depression severity levels. We also note that our training cannot serve as a substitute for a more comprehensive CBT course. For instance, researchers might explore additional skills such as training to help individuals reframe cognitive distortions (57) to neutralize their affective and psychosocial effects. In the context of many online sources spreading misinformation about CBT (61, 62), there is an urgent need to create and disseminate high-quality mental health materials to the public. Future studies could investigate a range of mental health resources to understand the limits of self-administered psychoeducation.

Here, we focused on interventions at the individual level, i.e. training individuals to recognize distorted content on social media. However, we might also intervene at the algorithmic level. Our findings reveal that individuals with depressive symptoms are more prone to interact with distorted content. One might counteract this effect by balancing the algorithmic curation of content that takes place on most social media platforms, e.g. by recommending different content to individuals that chose to avoid distorted content. Alternatively, algorithms might be developed using large language models or using communities to identify and flag the presence of distortions in social media content to protect against the potentially damaging effects of their diffusion (63, 64).

Similarly, interventions may be conceived at the collective level where individuals in a community, workplace, or school, may receive basic psychoeducational training in recognizing cognitive distortions in their interactions with other community members and possibly to respond in a “reframing” manner as shown effective by CBT. As members interact across a social network in their community, the effects of such minor interventions could be multiplied by network effects. In fact, research analyzing a large-scale linguistic corpus has revealed that the use of distorted language has increased sharply in recent decades (65). It might be possible for future researchers to conduct large-scale field studies where psychoeducation is widely distributed in social environments to study its impacts on individual behavior and social behavior of the community (66–68). Psychoeducational interventions like ours might be dispensed at scale on social media platforms to counteract the effects of distorted language across our society. This might help improve the quality of life even for subclinical populations (69) and marginalized and underserved communities (70) and bring about societal-scale changes.

## Methods

### Recruitment

A total of 1,000 individuals were recruited on MTurk using the platform Cloud Research from the United States. To ensure data quality, only CloudResearch-approved participants with an approval rating of more than 95% were recruited. We blocked duplicated IP addresses and only used individuals with verified worker country locations and blocked suspicious geocoded. The required sample size to detect a correlation of 0.15 with 0.8 power was 343 individuals (71). We therefore aimed to recruit 500 individuals per condition. Of the 1,000 individuals, 964 completed the experiment. Further, we employed two attention checks in our experiment and excluded the 116 participants who failed either of them. Ten additional participants were discarded for giving invalid entries for the willingness to pay questions. After all exclusions, we retained 838 participants. The gender distribution was 57.4% women, 39.86% men, 0.48% Trans men, 0.24% Trans women, 1.55% identified as other, and 0.48% preferred not to reveal their gender. The age distribution was 26.61% in the 18–30 range, 47.14% in the 31–45 range, 18.38% in the 46–60 range, and 7.88% in the 61–101 range. Of the total 838 participants, 418 were from the Interaction Before Training condition and 420 were from the Interaction After Training condition. Additional demographic details of the participants are presented in the Table S5.

### Data privacy and handling

For our experiment and subsequent analysis, we adhered to the protocol approved by the Institutional Review Board of Indiana University titled “Social Media and Cognitive Distortions—Wisdom of the Crowds” (#16984), which approved the experimental paradigm and data handling. Since our data contained information about self-reported health-related outcomes, we deidentified the data by assigning each individual a subject ID and removed potential identifying information like the Twitter handle and Amazon worker ID. All the data were stored on an encrypted IU server. This was accessible only to the members of the research team.

### Generation of stimuli

We generated a sample of sentiment-matched tweets that contained both distorted and nondistorted language on similar themes. We used the following three-step procedure (i) AI stimulus generation, (ii) clinical validation, and (iii) sentiment matching and validation. First, we prompted OpenAI’s Large Language Model—ChatGPT to generate tweets that contained cognitive distortions to avoid experimenter biases in themes and language. A sample prompt was “Now generate 10 more that contain cognitive distortions with similar sentence construction and sentiment.” We used two chat threads to increase the diversity of tweets. There was consensus within the research team that distorted and nondistorted statements contained similar themes, and the statements did not need additional contextual information to be understood. Using this approach, we generated a total of 120 tweets—60 distorted and 60 not distorted. Second, a licensed clinical psychologist (LL) inspected these tweets to validate them. Some of the tweets were modified so that they belonged to the correct category. From this set of validated tweets, a random subset of 30 tweets from each category was selected for the experiment, so that the entire procedure took about 30 min (the average experimental time was 25.7 min). Third, we used VADER, a well-validated sentiment analysis tool to rate the sentiment of each tweet on a −1 to 1 scale (72). These tweets were modified so that their VADER composite sentiment scores had similar distributions. We tested that the VADER composite scores had similar means ($M_{\mathrm{distorted}}=-0.296\;\text{SD}=0.37M_{\mathrm{nondistorted}}=-0.151\;\text{SD}=0.41,$  t(58)=1.45,P=0.1538 and similar distributions (Kolmogorov–Smirnov Test D(0)=0.2333,P=0.3929). The modifications were validated by a licensed clinical psychologist. This set of 60 tweets was used for the experiment.

### Training document

The training material utilized principles from cognitive behavioral therapy (CBT) interventions. It was specifically crafted to be both succinct and easily understood by individuals without a background in the field. Cognitive distortions were defined broadly as thoughts that “were not close to reality especially when too negative.” Three classes of distortions were described—jumping to conclusions, exaggerating, and being very rigid or strict. A representative example for each class was provided to aid comprehension. The document was written in straightforward and concise language to ensure clarity. There was no explicit description of the potentially destructive nature of cognitive distortions nor the link between cognitive distortions and mental illness. The full text of the training material is available in the Supplementary methods.

### Procedure

Participants provided their informed consent before participating in the experiment. Their Twitter handle and demographic information were collected before the main task. Participants were assigned to one of the two counter-balanced conditions—*Interaction Before Training* and *Interaction After Training* to study the impact of training on interaction. The assigned condition determined the order in which they saw the interaction and training-identification blocks.

During the training-identification block, participants first learned about cognitive distortions using the previously described training method. Following this, in the identification block, they were presented with the 30 randomly chosen tweets counter balanced across the two categories and asked to evaluate the probability (on a scale of 0–100) that each tweet contained a distortion. Participants provided their probability judgment by typing an integer between 0 and 100 into a text box. No feedback was given on their judgments. After providing their judgment, participants could interact with the tweet using the “like” or “retweet” buttons as described for the interaction block, using an interface similar to Twitter. In the interaction block, participants were presented with tweets generated from the remaining 30 randomly sampled stimuli also counter balanced across the two categories.

After the main task, we collected participant data on their mental health and social media use. The full details about the questions including question text are in the Supplementary methods. At the end of the experiment, participants were provided with resources for mental health support.

### Measuring depression levels

The PHQ-9 (39) was employed to evaluate current depression levels. The questionnaire asks individuals about the frequency of certain thoughts (such as hopelessness and suicidal ideation) and behaviors (such as weight gain and insomnia) during the preceding 2-week period. The responses from the nine questions are summed to create a final depression severity score (39). The full text of the questionnaire and scoring rule is presented in the Supplementary methods. This questionnaire has a high specificity and sensitivity for measuring depression (39–41). Its short length and validity when self-administered (73), made it possible to study depression in our large scale online experiment.

### Twitter Use Score

We used participants’ self-reported time spent on Twitter, active passive use, and willingness to pay judgment to create a composite TUS. The willingness to pay judgment was estimated by calculating the geometric mean of the bargain, expensive, and too expensive judgments. The active passive use score was recorded on a 0–100 scale, where 0 was passive and 100 was active. The daily time spent on social media was recorded in hours. We used the Boxcox transformation since the distribution was skewed. We then calculated the z-score for each of these scales so that each of the scales was centered and had the same SD, contributing equally to the score. The distribution of the individual questions and box-coxed transformed responses can be found in the Fig. S2. The TUS was the mean of these measures.

### Regression methods

We used a generalized mixed effects regression for our analysis. We tested for differences in (i) accuracy, (ii) liking, and (iii) retweeting. For the accuracy regression, we used behavioral data from the training-identification block. For the liking and retweeting regression, we used the behavioral data from the interaction block. We treated subject ID as a random effect. Since every participant saw a different randomly sampled set of stimuli, we controlled for this by treating every stimulus as a random effect. The Depressive Symptoms and TUS were centered and standardized before being used in the model. The models were fit using the Bound Optimization by Quadratic Approximation in the *glmer* method in the *lme4* R package.

To determine the best model of each dependent variable, we conducted a nested model comparison using five models

- base *y* ∼ block order * is distorted
- depression severity (alone) *y* ∼ block order * is distorted + *Depressive Symptoms*
- twitter use score (alone) *y* ∼ block order * is distorted + *Twitter Use Score*
- independent effects *y* ∼ block order * is distorted * Depressive Symptoms + block order * is distorted * Twitter Use Score
- full model: *y* ∼ block order * is distorted * Depressive Symptoms * Twitter Use Score

We conducted a likelihood ratio test to determine the best-fitting model. The interactive-independent model improved model fit compared to the linearly independent model. However, the full model did not improve the model fit across all of the dependent variables. The full list of coefficients and likelihood ratio test can be found in Supplementary Tables S1–S4. Hence, we report the results of the interactive-independent effects in the main paper. Additional analysis with the full model is reported in the Supplementary analysis. We also note that most of the coefficients are robust to the different assumptions made in the different models.

## Supplementary Material

pgaf068_Supplementary_Data

## Data Availability

Deidentified data and analysis code is publicly available on OSF https://osf.io/wjhv6/

## Previous Presentation

These results were previously presented at the Common Grounds Conference at Indiana University in April 2024.

## Preprints

A preprint of this article is published on OSF: https://osf.io/preprints/psyarxiv/47cqw_v1
