# Supplementary material for: One-shot intervention reduces online engagement with distorted content
Source: PNAS Nexus. 2025 Mar 4;4(3):pgaf068. doi: 10.1093/pnasnexus/pgaf068 (PMC11914320; doi:10.1093/pnasnexus/pgaf068)
Supplement: pgaf068_Supplementary_Data [file pgaf068_supplementary_data.pdf]

Supplementary Material for One shot intervention reduces online engagement with distorted content

Eeshan Hasan, Gunnar Epping, Lorenzo Lorenzo-Luaces, Johan Bollen, and Jennifer Trueblood
Indiana University

Correspondence concerning this article should be addressed to Eeshan Hasan (eehasan@iu.edu) or Jennifer S. Trueblood (jstruebl@iu.edu)

Supplemental Analysis

Performance Distribution

We present performance metrics for the identification task in Figure S1.

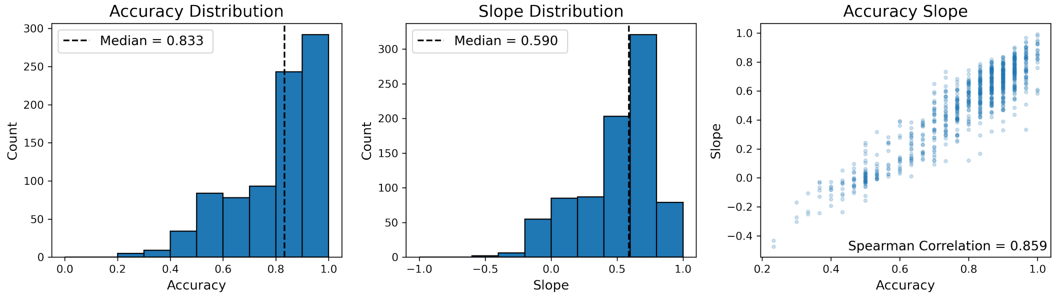

Figure S1

Performance metrics. The left panel depicts the accuracy distribution. The middle panel depicts the slope distribution. The right panel shows the relationship between accuracy and slope.

Twitter Questionnaire Distribution

We present the distribution of the responses to the Twitter Questionnaire S2. We observe that the distribution is skewed. We use the box-cox transformation to reduce the skew.

Relationship Plots

We plot the relationship between the different variables mentioned in the main paper. We present the relationship between the like and the retweet rate and performance, depressive symptoms and Twitter use score in S4. We present the relationship between the performance and depressive symptoms and Twitter use score in S3. We also present how the ratio of distorted (to total) liked and ratio of distorted (to total) retweeted depends on the accuracy.

Regressions

As described in the methods, we fit a set of nested generalized mixed effects regression models to predict accuracy (Table S1), liking (Table S2), and retweeting (Table S3). We observe that most of the coefficients have similar values across the models, indicating the robustness of our results to the different assumptions made in each model. Table S4 presents the results of a likelihood ratio test testing whether the more complex models had significantly better fits.

Table S1

Results of generalized mixed effects regression predicting accuracy (N=838). This analysis shows that the coefficients reported in the main paper are robust to different assumptions made in different models.

	Dependent variable: Accuracy				
	Base	Depression Severity	Twitter Use	Independent	Full
	(1)	(2)	(3)	(4)	(5)
Age	1.163* [1.025,1.319]	1.130 [0.993,1.286]	1.089 [0.964,1.229]	1.069 [0.944,1.210]	1.069 [0.943,1.212]
Woman	1.338*** [1.140,1.570]	1.373*** [1.167,1.615]	1.249** [1.071,1.457]	1.274** [1.089,1.489]	1.277** [1.091,1.496]
Non-Binary	1.722* [1.046,2.834]	1.862* [1.125,3.083]	1.674* [1.039,2.698]	1.774* [1.094,2.875]	1.788* [1.096,2.918]
Interaction After Training	1.035 [0.867,1.235]	1.026 [0.857,1.228]	0.960 [0.805,1.145]	0.941 [0.787,1.125]	0.927 [0.773,1.111]
Is Distorted	0.395*** [0.273,0.570]	0.379*** [0.262,0.549]	0.355*** [0.245,0.512]	0.342*** [0.236,0.495]	0.332*** [0.228,0.482]
Depression Severity		0.199*** [0.119,0.334]		0.205*** [0.124,0.339]	0.330*** [0.193,0.563]
Twitter Use Score			0.148*** [0.095,0.230]	0.152*** [0.097,0.237]	0.199*** [0.126,0.315]
Interaction After Training:Is Distorted	1.029 [0.895,1.184]	1.034 [0.897,1.193]	1.076 [0.930,1.245]	1.093 [0.943,1.267]	1.097 [0.945,1.274]
Interaction After Training: Depression Severity		1.036 [0.492,2.180]		1.528 [0.737,3.172]	1.099 [0.512,2.358]
Is Distorted:Depression Severity		6.678*** [4.450,10.022]		6.485*** [4.305,9.769]	4.090*** [2.618,6.388]
Interaction After Training: Is Distorted:Depression Severity		1.054 [0.582,1.909]		0.789 [0.431,1.443]	1.069 [0.563,2.027]
Interaction After Training:Twitter Use Score			0.567 [0.298,1.078]	0.657 [0.343,1.259]	0.549 [0.282,1.071]
Is Distorted:Twitter Use Score			3.990*** [2.790,5.706]	3.832*** [2.672,5.496]	2.835*** [1.953,4.114]
Depression Severity:Twitter Use Score					0.001*** [0.000,0.008]
Interaction After Training: Is Distorted:Twitter Use Score			1.619 [0.960,2.729]	1.326 [0.782,2.249]	1.600 [0.930,2.752]
Interaction After Training: Depression Severity:Twitter Use Score					154.808*** [8.714,2750.340]
Is Distorted:Depression Severity: Twitter Use Score					26351.224*** [5015.565,138446.424]
Interaction After Training:Is Distorted: Depression Severity:Twitter Use Score					0.001*** [0.000,0.007]
Constant	5.477*** [3.709,8.088]	5.957*** [4.017,8.834]	7.201*** [4.906,10.572]	7.697*** [5.222,11.345]	7.945*** [5.369,11.756]
Observations	25,140	25,140	25,140	25,140	25,140
Log Likelihood	-10,847	-10,766.0	-10,740.2	-10,674	-10,592.7
Akaike Inf. Crit.	21,713.6	21,558.1	21,506.5	21,382.7	21,227.4
Bayesian Inf. Crit.	21,786.7	21,663.8	21,612.2	21,520.9	21,398.2

*$p<0.05$; **$p<0.01$; ***$p<0.001$

Note: Items and individuals were treated as random effects. The 95% confidence intervals are presented below the coefficients.

Table S2

Results of generalized mixed effects regression predicting liking (N=838). This analysis shows that the coefficients reported in the main paper are robust to different assumptions made in different models.

	Dependent variable: Like Rate				
	Base	Depression Severity	Twitter Use	Independent	Full
	(1)	(2)	(3)	(4)	(5)
Age	1.061	1.191*	1.112	1.239*	1.240*
	[0.897,1.255]	[1.008,1.408]	[0.942,1.314]	[1.050,1.462]	[1.051,1.462]
Woman	1.148	1.047	1.209	1.097	1.100
	[0.926,1.422]	[0.848,1.293]	[0.977,1.495]	[0.890,1.352]	[0.893,1.355]Bas
Non-Binary	1.328	0.956	1.346	0.991	1.000
	[0.694,2.540]	[0.504,1.811]	[0.709,2.555]	[0.528,1.861]	[0.533,1.876]
Interaction After Training	1.000	1.018	1.018	1.034	1.055
	[0.807,1.238]	[0.826,1.254]	[0.824,1.259]	[0.841,1.272]	[0.857,1.299]
Is Distorted	0.242***	0.227***	0.227***	0.212***	0.210***
	[0.153,0.385]	[0.143,0.361]	[0.143,0.361]	[0.133,0.336]	[0.132,0.335]
Depression Severity		1.612		1.613	1.638
		[0.866,2.999]		[0.874,2.976]	[0.890,3.016]
Twitter Use Score			1.709	1.742*	1.797*
			[0.983,2.968]	[1.016,2.987]	[1.049,3.079]
Interaction After Training: Is Distorted	0.544***	0.515***	0.573***	0.553***	0.546***
	[0.471,0.628]	[0.444,0.598]	[0.495,0.664]	[0.475,0.644]	[0.468,0.636]
Interaction After Training: Depression Severity		2.027		2.023	2.044
		[0.833,4.936]		[0.837,4.893]	[0.848,4.928]
Is Distorted:Depression Severity		7.112***		7.241***	7.444***
		[4.809,10.516]		[4.883,10.737]	[5.008,11.067]
Interaction After Training: Is Distorted:Depression Severity		1.292		0.986	0.876
		[0.708,2.358]		[0.536,1.811]	[0.474,1.619]
Interaction After Training: Twitter Use Score			0.700	0.600	0.626
			[0.316,1.551]	[0.275,1.309]	[0.286,1.369]
Is Distorted:Twitter Use Score			3.577***	3.640***	3.857***
			[2.497,5.124]	[2.530,5.238]	[2.655,5.603]
Depression Severity:Twitter Use Score					0.137
					[0.015,1.243]
Interaction After Training: Is Distorted:Twitter Use Score			1.601	1.211	0.932
			[0.930,2.758]	[0.697,2.102]	[0.523,1.659]
Interaction After Training: Depression Severity:Twitter Use Score					0.807
					[0.029,22.605]
Is Distorted:Depression Severity: Twitter Use Score					0.497
					[0.117,2.118]
Interaction After Training:Is Distorted: Depression Severity:Twitter Use Score					29.398**
					[2.833,305.084]
Constant	0.464**	0.391***	0.405***	0.348***	0.347***
	[0.280,0.770]	[0.237,0.647]	[0.244,0.672]	[0.210,0.574]	[0.211,0.573]
Observations	25,140	25,140	25,140	25,140	25,140
Log Likelihood	-11,227.5	-11,112.0	-11,158.9	-11,054.8	-11,047.9
Akaike Inf. Crit.	22,472.9	22,250.0	22,343.8	22,143.7	22,137.9
Bayesian Inf. Crit.	22,546.1	22,355.8	22,449.5	22,281.9	22,308.6

*p<0.05; **p<0.01; ***p<0.001

Note: Items and individuals were treated as random effects. The 95% confidence intervals are presented below the coefficients.

Table S3

Results of generalized mixed effects regression predicting retweeting (N=838). This analysis shows that the coefficients reported in the main paper are robust to different assumptions made in different models.

	Dependent variable: Retweet Rate				
	Base	Depression Severity	Twitter Use	Independent	Full
	(1)	(2)	(3)	(4)	(5)
Age	1.002<br>[0.798,1.258]	1.177<br>[0.938,1.476]	1.116<br>[0.894,1.393]	1.288^{*}<br>[1.033,1.607]	1.289^{*}<br>[1.034,1.608]
Woman	0.858<br>[0.642,1.148]	0.756<br>[0.568,1.007]	0.947<br>[0.713,1.256]	0.832<br>[0.629,1.100]	0.833<br>[0.630,1.101]
Non-Binary	0.867<br>[0.358,2.095]	0.567<br>[0.237,1.355]	0.930<br>[0.396,2.187]	0.627<br>[0.269,1.458]	0.648<br>[0.278,1.508]
Interaction After Training	0.946<br>[0.709,1.260]	0.965<br>[0.729,1.277]	1.031<br>[0.779,1.364]	1.045<br>[0.794,1.374]	1.065<br>[0.809,1.403]
Is Distorted	0.406^{***}<br>[0.268,0.616]	0.372^{***}<br>[0.245,0.564]	0.362^{***}<br>[0.238,0.549]	0.326^{***}<br>[0.214,0.496]	0.318^{***}<br>[0.209,0.484]
Depression Severity		4.399^{***}<br>[1.925,10.051]		4.187^{***}<br>[1.879,9.328]	4.191^{***}<br>[1.880,9.342]
Twitter Use Score			6.392^{***}<br>[3.088,13.232]	6.189^{***}<br>[3.037,12.611]	6.163^{***}<br>[3.017,12.589]
Interaction After Training:Is Distorted	0.436^{***}<br>[0.362,0.524]	0.405^{***}<br>[0.332,0.494]	0.435^{***}<br>[0.356,0.530]	0.424^{***}<br>[0.343,0.524]	0.434^{***}<br>[0.351,0.537]
Interaction After Training:<br>Depression Severity		1.229<br>[0.376,4.022]		1.123<br>[0.353,3.566]	1.158<br>[0.364,3.682]
Is Distorted:Depression Severity		4.735^{***}<br>[2.948,7.607]		5.136^{***}<br>[3.176,8.306]	5.680^{***}<br>[3.488,9.252]
Interaction After Training:<br>Is Distorted:Depression Severity		1.661<br>[0.765,3.607]		1.159<br>[0.526,2.552]	0.991<br>[0.437,2.248]
Interaction After Training:<br>Twitter Use Score			0.405<br>[0.143,1.151]	0.346^{*}<br>[0.124,0.968]	0.382<br>[0.135,1.077]
Is Distorted:Twitter Use Score			3.068^{***}<br>[1.982,4.749]	3.326^{***}<br>[2.136,5.180]	4.017^{***}<br>[2.514,6.418]
Depression Severity:Twitter Use Score					0.910<br>[0.051,16.184]
Interaction After Training:<br>Is Distorted:Twitter Use Score			2.761^{**}<br>[1.351,5.644]	2.046<br>[0.993,4.217]	1.551<br>[0.705,3.412]
Interaction After Training:<br>Depression Severity:Twitter Use Score					0.112<br>[0.001,9.016]
Is Distorted:Depression Severity:<br>Twitter Use Score					0.129^{*}<br>[0.024,0.686]
Interaction After Training:Is Distorted:<br>Depression Severity:Twitter Use Score					21.243<br>[0.910,495.766]
Constant	0.105^{***}<br>[0.057,0.191]	0.083^{***}<br>[0.046,0.151]	0.077^{***}<br>[0.043,0.140]	0.064^{***}<br>[0.035,0.115]	0.064^{***}<br>[0.035,0.115]
Observations	25,140	25,140	25,140	25,140	25,140
Log Likelihood	-7,466.1	-7,403.7	-7,402.7	-7,345.8	-7,342.0
Akaike Inf. Crit.	14,950.2	14,833.4	14,831.3	14,725.7	14,726.0
Bayesian Inf. Crit.	15,023.4	14,939.2	14,937.0	14,863.9	14,896.7

$^{*}p<0.05$; $^{**}p<0.01$; $^{***}p<0.001$

Note: Items and individuals were treated as random effects. The 95% confidence intervals are presented below the coefficients.

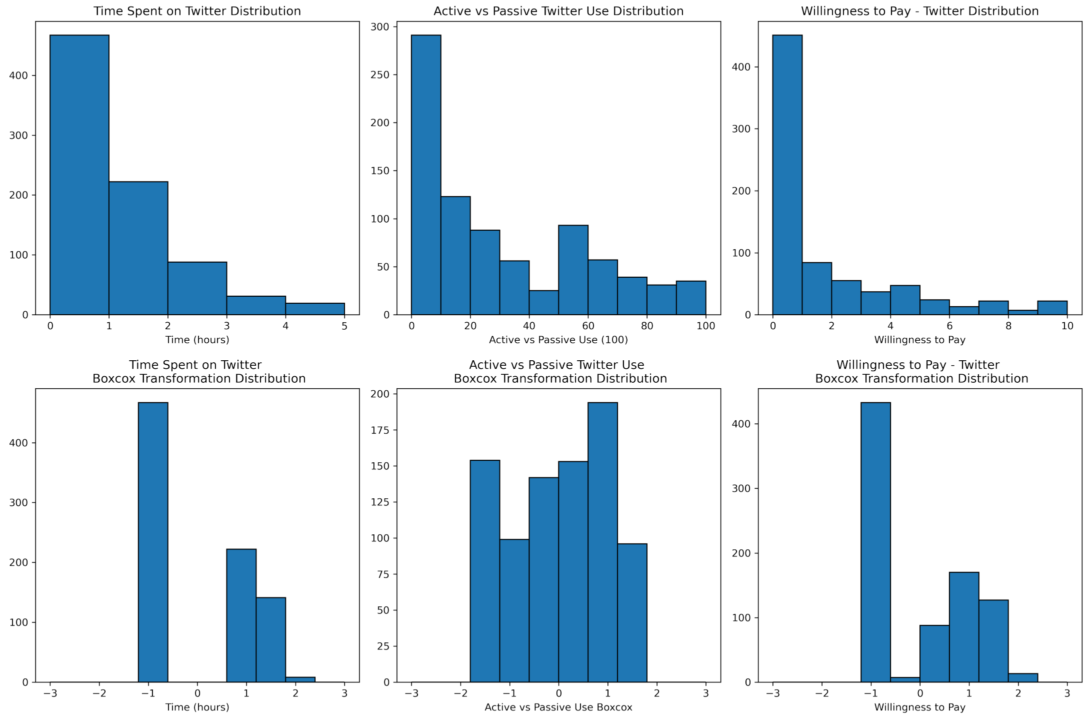

Figure S2

In the top panels, we show the distribution of responses to the Twitter questionnaire. In the bottom panels, we show the z-score normalized box-cox transformed versions of these distributions.

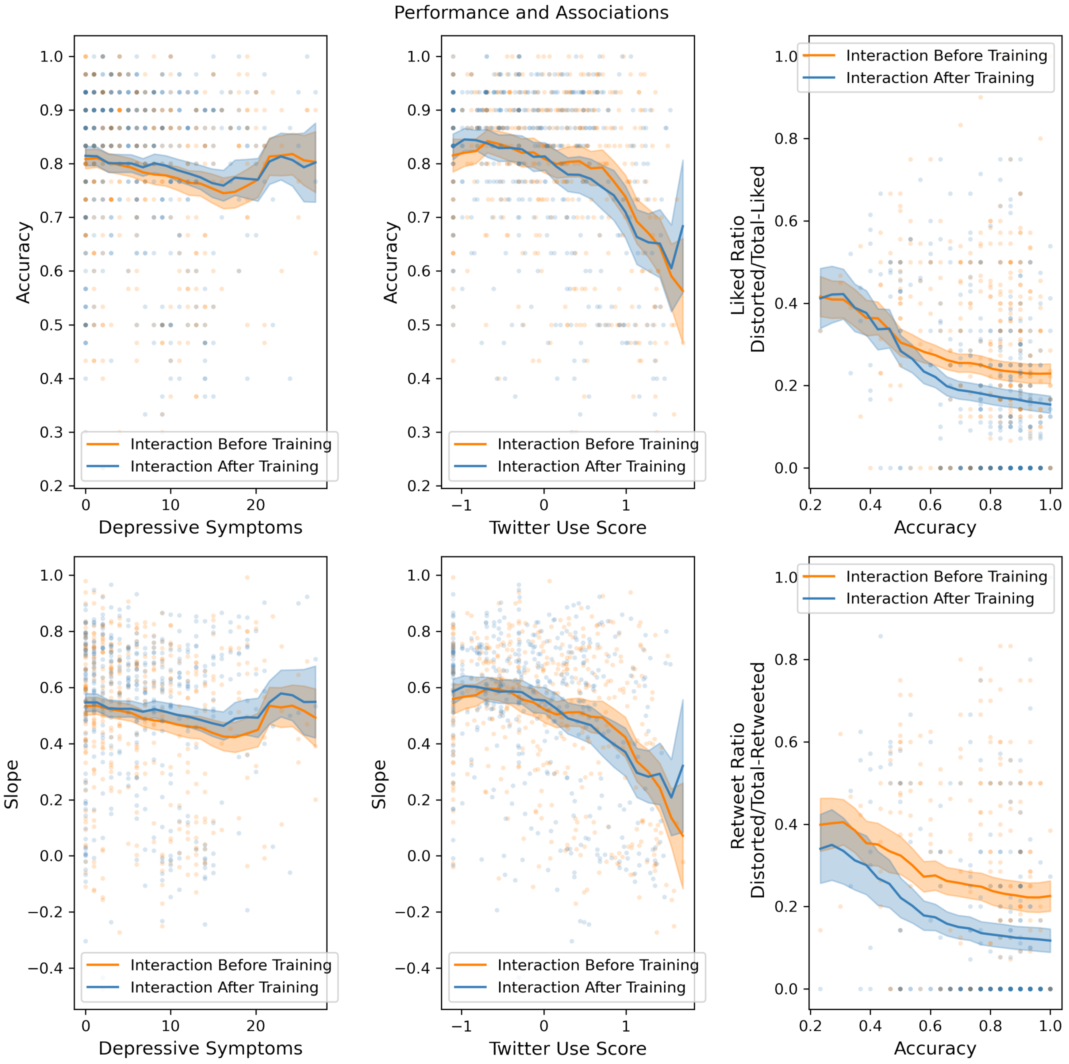

Figure S3

The relationship between performance and depressive symptoms and twitter use score.

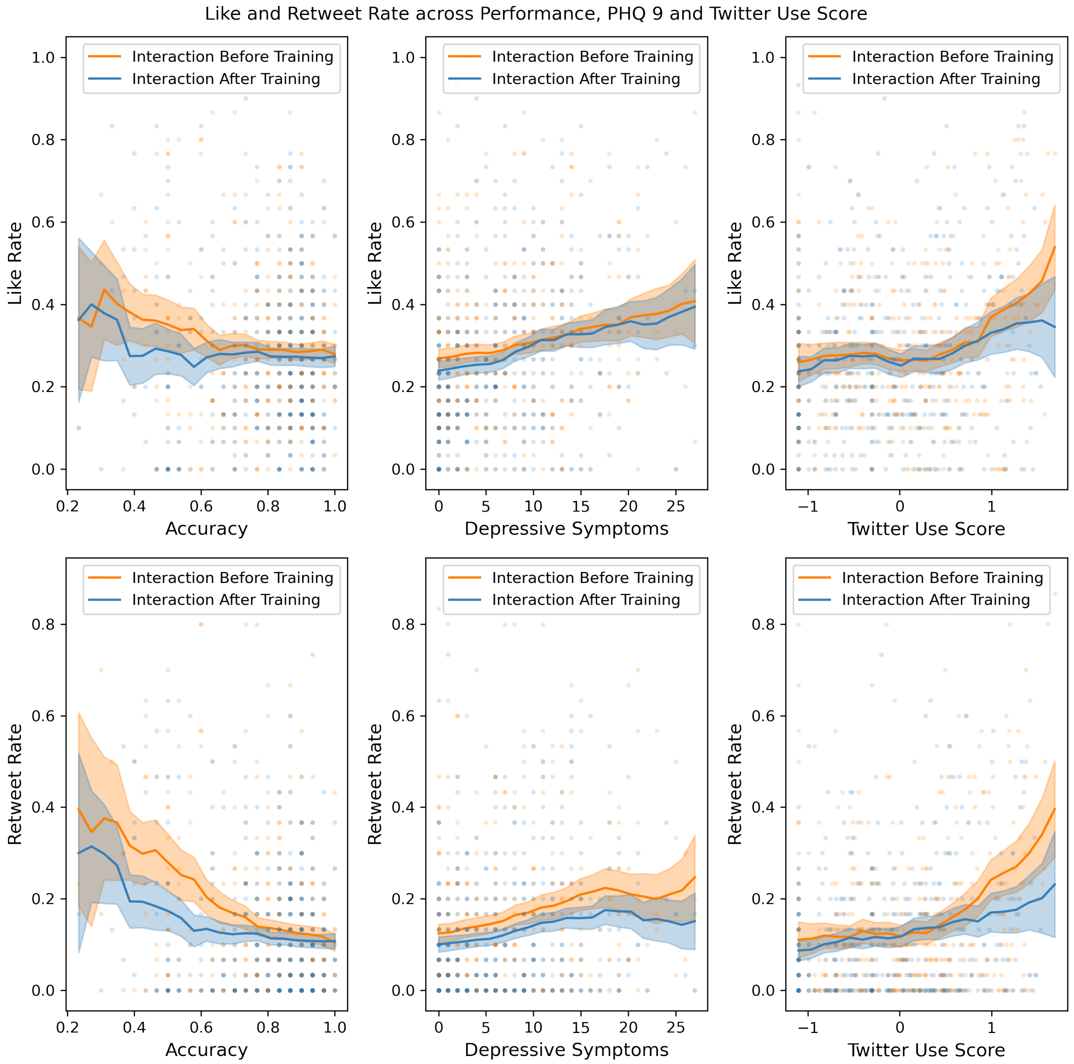

Figure S4

The relationship between like and retweet rate and performance, depressive symptoms and Twitter use score.

Table S4

Nested Model Comparison: Likelihood Ratio Test

	Accuracy Model Comparison	Chisq	p.value
1	Base vs Depression Severity	163.49	0.00
2	Base vs Twitter Use	215.07	0.00
3	Depression Severity vs Independent	183.42	0.00
4	Twitter Use vs Independent	131.84	0.00
5	Independent vs Full	163.24	0.00
	Like Model Comparison		
1	Base vs Depression Severity	230.91	0.00
2	Base vs Twitter Use	137.18	0.00
3	Depression Severity vs Independent	114.37	0.00
4	Twitter Use vs Independent	208.10	0.00
5	Independent vs Full	13.81	0.01
	Retweet Model Comparison		
1	Base vs Depression Severity	119.18	0.00
2	Base vs Twitter Use	126.77	0.00
3	Depression Severity vs Independent	114.59	0.00
4	Twitter Use vs Independent	107.00	0.00
5	Independent vs Full	7.90	0.10

Supplemental Methods

We provide supplemental information regarding our methods in the following sections.

Demographics

We report the demographics of the main experiment in Table S5.

Prompts for stimuli generation

The following prompts in two chats (labeled A and B below) were used to generate distorted content using ChatGPT. The link to the transcripts of the chat can be found here. In the first chat thread, the prompts were the following:

1A Generate 10 tweets with negative sentiment that do not have cognitive distortions

2A Generate 10 tweets that are similar to the ones above in sentence construction and sentiment but have cognitive distortions

3A Thank you. Most of these sentences have I's in them. Can you generate ones that have cognitive distortions but do not necessarily start with I

4A Now generate 10 tweets that have negative sentiment but do not have cognitive distortions

5A Now generate 10 tweets with cognitive distortions

6A Now generate 10 that do not contain cognitive distortions with similar language

7A Now generate 10 more that contain cognitive distortions that have similar sentence construction and sentiment

8A Now generate 10 tweets that do not contain cognitive distortions with different themes and topics from the ones mentioned above but with negative sentiment

9A Now generate 10 more that contain cognitive distortions that have similar sentence construction and sentiment

10A Now generate 10 tweets that do not contain cognitive distortions with different themes and topics from the ones mentioned above

The link to the transcript of the chat can be found here. In the second chat thread, the prompts were the following.

1B Generate 10 tweets containing cognitive distortions that do not have a positive sentiment and do not begin with I statements

2B Generate 10 more tweets with more words

3B Generate 10 more similar tweets with more words and fewer I statements

Gender Identity	Count	Percentage
Woman	481	57.40 %
Man	334	39.86 %
Trans man	4	0.48 %
Trans woman	2	0.24 %
Other	13	1.55 %
Prefer not to say	4	0.48 %
Race		
White	631	75.30 %
Black	93	11.10 %
Multiracial	41	4.89 %
Asian	38	4.53 %
Native American or Pacific Islander	7	0.84 %
Other	18	2.15 %
Prefer not to say	10	1.19 %
Ethnicity		
Non-Hispanic	745	88.90 %
Hispanic	83	9.90 %
Prefer not to say	10	1.19 %
Age		
18 - 30	223	26.61 %
31 - 45	395	47.14 %
46 - 60	154	18.38 %
61 - 101	66	7.88 %
PHQ 9 Class		
1. No/Minimal Depression (PHQ-9 Score: 0-4)	373	44.5 %
2. Mild Depression (PHQ-9 Score: 4-9)	203	24.2 %
3. Moderate Depression (PHQ-9 Score: 10-14)	153	18.3%
4. Moderately Severe Depression (PHQ-9 Score: 14-19)	70	8.4 %
5. Severe Depression (PHQ-9 Score: 20-27)	39	4.7 %

Table S5

Demographics of the participants in our experiment after the exclusions.

4B Generate 10 more similar tweets without cognitive distortions

5B Generate 10 new tweets that do not contain cognitive distortions and have a gloomy sentiment

6B These tweets do contain cognitive distortions, please generate similar tweets with more words that do not contain cognitive distortions

7B Generate 10 more tweets that vary more in topics and sentence structure

8B Generate 10 more tweets that are similar but do contain cognitive distortions

9B Generate 10 more tweets without cognitive distortions

10B vary the sentence structure more

11B try again

12B vary the sentence structure more

Stimuli

In this subsection, we provide the list of distorted and non-distorted stimuli used in our experiment.

Below is the list of distorted stimuli used in our experiment:

1. "I'll never find love. I'm just not good enough for anyone."
2. "Bad things happen because the universe is cruel and punishing."
3. "I'm always overwhelmed because I can't handle anything. I'm weak."
4. "People are always talking about me behind my back. I must be a terrible person."
5. "I'll never be able to handle the morning rush hour. I'm just not a good enough driver."
6. "I'm never going to be able to like horror movies. I'm just too scared."
7. "I hate this weather because I can't handle it. I'm weak."
8. "The city is bustling and overwhelming, I can't handle it anymore."
9. "Being alone is the norm. No one will ever understand us."
10. "I'm always the last one to know. No one ever tells me anything."
11. "Life is always unfair to me. I never get a break."
12. "I can't handle this stress. I'm going to crumble."
13. "I always ruin things. I'm just not cut out for public speaking."
14. "I always fail. I'm just not meant for success."
15. "Politics are divisive and there's no hope for a better future."
16. "It's impossible to enjoy anything. Everything is always a disappointment."
17. "Finances are tight and I'll never be able to get ahead."
18. "It's difficult to trust anyone, there's always a chance of betrayal."
19. "The world is constantly filled with negativity, it's hard to find any positivity."
20. "People always disappoint me. No one is trustworthy."
21. "Negative thoughts are hard to shake, it's difficult to break out of this cycle."

22. "People are often selfish and only think of themselves, it's hard to find genuine connections."
23. "Work deadlines are approaching quickly and I'm never going to catch up."
24. "It seems like bad luck is always around the corner, it's hard to find success."
25. "Navigating relationships can be complicated, I'm always going to be alone."
26. "I'm a total failure. Nothing I do is ever good enough."
27. "People are always talking about me behind my back. No one really likes me."
28. "I'm never going to get this project right. I'm just not smart enough."
29. "Everyone else has it easy. Life is always harder for us."
30. "This problem seems unsolvable, there's no way to overcome it."

Below is the list of non-distorted stimuli used in our experiment:

1. "I was sad to hear about the passing of someone I knew."
2. "The new phone I just bought is disappointing. It's slow, the battery drains quickly and the camera is subpar."
3. "I've been feeling very busy lately and it's taking a toll on me."
4. "I'm feeling a lot of stress with all my responsibilities. It's a lot to manage."
5. "I just got stuck in traffic for hours and now I'm running late for my meeting."
6. "I'm not a fan of cooking. I just don't have the patience for it."
7. "I'm really not a fan of horror movies. They scare me too much."
8. "City life presents its fair share of difficulties, but carving out time for self-care is a must for mental and emotional well-being."
9. "My neighbor's dog is barking non-stop. I can't get any peace and quiet."
10. "I'm not happy with my recent performance at work. I want to do better."
11. "I'm feeling frustrated with the current political climate and all the division."
12. "I had a tough day today. I'm feeling exhausted and drained."
13. "I don't like public speaking. I get so nervous and it's just not my thing."
14. "I didn't do well on my recent job opportunity. It was a disappointing outcome."
15. "Political views can be divisive, but respecting diverse opinions and working towards mutual understanding is important for a better future."

16. "This weather has been stormy. It's been raining non-stop for days and I do not like it."
17. "Managing finances can be a challenge, but creating a budget and seeking advice from financial experts can provide stability."
18. "Trusting people is difficult, but it's worth the effort for relationships."
19. "Life can be challenging, but it's important to not dwell on the negative too much."
20. "I'm feeling frustrated with the current political situation. There's a lot of division."
21. "It's normal to have negative thoughts, but it's important to challenge them."
22. "People can be unpredictable, but it's important to surround yourself with supportive relationships."
23. "Work can be demanding, with deadlines looming. To handle the workload, prioritize tasks and seek support from coworkers."
24. "Bad luck is a part of life, but it's important to persevere."
25. "Building and nurturing relationships requires effort, but staying open-minded and honing communication skills lead to deeper connections."
26. "This project is really challenging. I'm struggling to keep up with the workload."
27. "I'm sad to hear about the passing of someone I knew."
28. "I've been having a difficult time with this project. It's been challenging for me."
29. "The concert I went to last night was a letdown. The sound was off and the artist was not as good as I expected."
30. "Problems can seem overwhelming, but taking small steps can move things forward."

Training Document

The following is the text of the training document used to teach individuals about cognitive distortions:

Cognitive Distortions

All of us have thousands of thoughts every day. These thoughts come to us as words, sentences, or images that can pop into our head when we are doing things.

Many of these thoughts are accurate. That is, they are close to reality. Many of our thoughts, however, are "distorted." We call thoughts “distorted” when they are not close to reality, especially when they are too negative.

A lot of times distorted thoughts take the form of:

- *Jumping to conclusions:* Jumping to conclusion is assuming that you know what is going to happen based on a little information. For example, if a friend tells you they want to talk to you, jumping to conclusions would be assuming they are going to give you bad news even though you have no reason to think that.

- *Exaggerating:* Exaggerating means thinking of something as worse than it really is. For example, suppose you have to call the internet company because you are having issues with your internet connection, an example of exaggeration would be thinking "this is terrible."

- *Being very rigid or strict:* Being very strict or rigid means not being willing to change or adapt to situations. For example, if someone told you they would e-mail you are 12:00PM and they actually end up e-mailing you at 12:15PM, being very rigid or strict would be thinking "they should have e-mailed me at 12:00PM" even if the issue was not time sensitive.

Attention Checks

The questions below were used to test if the participants were attentive during the experiment.

- **Attention Check 1:** I work 14 months in a year.
  Options: Yes, this is true of me; No, this is not true of me.
  Correct Answer: No, this is not true of me.

- **Attention Check 2:** I have never used a computer.
  Options: Yes, this is true of me; No, this is not true of me.
  Correct Answer: No, this is not true of me.

PHQ 9 Questionnaire

The PHQ9 Questionnaire is a short questionnaire that is used to assess depression. Specifically, the questions are:

1. Little interest or pleasure in doing things

2. Feeling down, depressed, or hopeless

3. Trouble falling or staying asleep, or sleeping too much

4. Feeling tired or having little energy

5. Poor appetite or overeating

6. Feeling bad about yourself or that you are a failure or have let yourself or your family down

7. Trouble concentrating on things, such as reading the newspaper or watching television

8. Moving or speaking so slowly that other people could have noticed. Or the opposite being so figety or restless that you have been moving around a lot more than usual

9. Thoughts that you would be better off dead or of hurting yourself

For each question, individuals can rate the frequency at which they have felt each of these symptoms. They can respond using one of the four options (i) Not at all (ii) Several days (iii) More than half the days (iv) Nearly everyday. Each response is given a score of 0, 1, 2 and 3 respectively based on the frequency. These scores are added up to produce an aggregate score ranging from 0-27.

CBT Exposure

The following questions were used to assess if the individuals had been exposed to CBT in the past. They could respond (i) Yes (ii) No (iii) I am not sure.

- *Have you ever seen a mental health professional like a counselor, psychologist, or social worker for any kind of psychotherapy, "talk therapy," or counseling?*
- *Have you ever seen a mental health professional to do cognitive-behavioral therapy, sometimes known as CBT, or cognitive therapy (CT)?*

Diagnoses Questionnaire

Participants were asked if they were ever diagnosed with the following mental illness. For each question, participants had the following response options - (i) Yes (ii) No (iii) I don't know (iv) No, but I should be.

- Depression
- Insomnia
- Somatic symptom disorder (or chronic pain)
- A specific phobia
- Social anxiety disorder
- Panic disorder
- Post-traumatic stress disorder (PTSD)
- Generalized anxiety disorder
- Agoraphobia
- Alcohol use disorder or alcoholism
- Other substance use disorder (e.g., cannabis)
- Mania or bipolar disorder

Social Media Questionnaire

Participants were asked a series of questions about their social media usage patterns across various platforms.

Platforms List

Participants were asked about the following social media platforms:

- *Twitter*
- *Facebook*
- *Instagram*
- *TikTok*
- *Snapchat*
- *Reddit*
- *YouTube*

Willingness to Pay

Instructions: *Many people are quite attached to their social media accounts. How much are they worth to you? For each of the following social media platforms, suppose that they began requiring a monthly subscription. As a result, to keep your account and continue using each platform, you must now pay a monthly fee. At what price do you perceive that the social media platform is beginning to get expensive, so that it is not out of the question, but you would have to give some thought to buying it?*

- *At what price would you consider the social media platform to be a bargain - a great buy for the money?*
- *At what price do you begin to perceive the social media platform as so expensive that you would not consider buying it?*
- *At what price do you begin to perceive the social media platform as so expensive that you would not consider buying it?*

Time Spent

On average, how much time per day are you on this social media platform?

Active Passive Use

What proportion of the time are you actively using this social media platform (as opposed to passively)?

Cognitive Distortion Production Task

Participants were prompted to generate sentences that demonstrate the presence and absence of a distortion. The following are examples of the prompts that we used:

- *Create a tweet that has a cognitive distortion.*
- *Create a tweet that does not have a cognitive distortion.*
